# Editorial: Women in nutrition and metabolism

**DOI:** 10.3389/fnut.2023.1181638

**Published:** 2023-03-28

**Authors:** Clare M. Reynolds, Teodora Handjieva-Darlenska, Julie-Anne Nazare

**Affiliations:** ^1^School of Public Health, Physiotherapy and Sports Science, Conway Institute, Institute of Food and Health, University College Dublin, Dublin, Ireland; ^2^Faculty of Medicine, Medical University, Sofia, Bulgaria; ^3^CarMeN Laboratory, Centre de Recherche en Nutrition Humaine Rhône-Alpes, Univ-Lyon, Hospices Civils de Lyon, CENS, Université Claude Bernard Lyon 1, Pierre-Bénite, France

**Keywords:** women, research, metabolism, reproduction, agriculture

Despite a continuing growth in the number of women entering undergraduate, masters and postgraduate level courses, females are hugely underrepresented in research-based careers. A recent UNESCO (2015–2018) report has highlighted that only one third of researchers worldwide are female, this drops to one quarter at the full professorship level. In addition, women are credited less in scientific publications than males, with implications for the retention of female researchers and promotion to higher academic levels (1). The recent COVID-19 crisis has aggravated this situation with documented falls in both publication rates and grant success in female researchers (2). There also remains a significant disparity in research involving both male and female subjects (3). Historically many studies have excluded female participants, be it human intervention trials or basic pre-clinical research, resulting in higher likelihood of misdiagnoses and inappropriate or ineffective treatments. However, this situation is slowly changing with various national and European calls requiring justification for studies that do not address gender in their research design. This Research Topic in Frontiers in Nutrition, “*Women in nutrition and metabolism”* highlights the diversity of research from female researchers in the Nutrition and Metabolism field.

This includes basic science research examining osteoporosis and immunomodulation in mice exposed to high fructose diets (Tsai et al.) and the role of different zinc sources on mouse myofibroblasts growth and piglet carcass traits (Zhang et al.). This issue also includes studies evaluating the efficacy of multidisciplinary obesity outpatient service on BMI in children and adolescents (Wyse et al.) and dietary behavior and compliance to national nutrition guidelines in a cohort of Type I diabetic patients (Pancheva et al.). The final study included in this Research Topic systematically reviewed the literature in relation to artificial sweetener exposure on male fertility outcomes (Kearns et al.).

Tsai et al. examine the impact of whole-body vibration as an exercise mode on excessive fructose consumption in ovariectomized mice which were used as a model of post-menopausal women. Vibration training is a passive exercise intervention which is thought to improve osteoporosis, weight gain and glycemic control without the risk of falls, fractures and joint problems in elderly participants. The authors examined the potential of this form of therapy on ameliorating high fructose-induced metabolic dysfunction and bone loss in ovariectomized mice. They found that a high fructose diet exacerbates ovariectomy induced metabolic inflammation but that this can be reduced when whole body vibration therapy is applied. However, this form of exercise did not impact the risk of osteoporosis. There are a lot of misconceptions about menopause particularly in relation to nutrition and exercise management. Given the links between menopause and changes in metabolic health and bone density, basic science studies are key to tailoring nutrition and diet advice in this population.

While many nutrition based basic science studies focus on human interventions or pre-clinical models, the role of nutrition in relation to agriculture is often overlooked. Zhang et al. demonstrated that supplementation with difference sources of zinc and impact growth in both myoblast cells lines and growth performance in growing-finishing pigs. They showed that methionine-chelated zinc and glycine -chelated zinc supplementation was more effective in relation to enhancing muscle growth through increasing bioavailability of multiple micronutrients. These findings were then linked to increased expression of cell cycle, OXPHOS and mTOR pathways. This increase in growth performance with methionine-chelated zinc has important implications for the formulation of animal nutrition products.

The broad scope of research carried out by women in the nutrition and metabolism domain is reflected in the diverse topics covered in this Research Topic. Pancheva et al. examine the dietary compliance and adherence to the National Nutrition Guidelines in a cohort of Bulgarian Type 1 Diabetic patients. This case-control study in patients with long-standing Type 1 Diabetes demonstrated sex-specific effects in relation to macronutrient intakes using a 24 h recall as well as a food frequency questionnaire. Interestingly, while the participants fell short of the national nutrition guidelines, they typically had a heathier diet than the general population.

Metabolism research was also reflected in the study by Wyse et al. They examined the impact of a family-based, multidisciplinary child and adolescent obesity outpatient service on BMI. The combination of support from a physiotherapist, dietician and psychologist to develop a personalized plan to tackle obesity in children was associated with reductions in standardized BMI over a 12-year period in an Irish population. They found that BMI reduction was greater in younger children and in children who had longer treatment duration despite extensive between subject variability. This underscores the importance of multidisciplinary approaches in obesity management and highlights the multifactorial etiology of obesity which is driven by genetic, social, pathological and life-style related factors.

Lastly, Kearns et al. present a systematic review and narrative synthesis of the literature on the impact of non-caloric sweeteners in male fertility. This review focused on rodent models given the lack of literature on human subjects. Despite a firm conclusion on the effect of sweeteners on male fertility there were evidence of dose dependent effects of non-caloric sweeteners. Potential mechanisms of action that were highlighted include oxidative stress, androgenic effects and the activation of taste receptors. Issues such as study duration, type of sweetener, assessment method and dose used all contributed to significant heterogeneity. It is clear from this review that further studies, particularly in humans are required to determine the impact of sweeteners on male fertility.

Overall, this special topic in Frontiers Nutrition and Metabolism provides relevant examples of the diversity, originality and quality of research carried out by female researchers in this field, supporting continuous and on-going active involvement of women in research and the importance for further initiatives to promote young women researchers.

## Author contributions

CR wrote the editorial. J-AN and TH-D edited the editorial. All authors contributed to the article and approved the submitted version.
